# Safety Toxicology Study of Reassortant Mopeia–Lassa Vaccine in Guinea Pigs

**DOI:** 10.3390/futurepharmacol5020026

**Published:** 2025-05-31

**Authors:** Bradley S. Wahle, Peter Pushko, Katie Albanese, Dylan M. Johnson, Irina Tretyakova, Igor S. Lukashevich, Thomas Rudge

**Affiliations:** 1 MRIGlobal, Kansas City, MO 64110, USA; 2 Medigen, Inc., Frederick, MD 21701, USA; 3 Battelle, West Jefferson, OH 43162, USA; 4 Sandia National Laboratory, Department of Biotechnology & Bioengineering, Livermore, CA 94550, USA; 5 Department of Pharmacology and Toxicology, Center for Predictive Medicine for Biodefense and Emerging Infectious Diseases, University of Louisville, Louisville, KY 40292, USA

**Keywords:** toxicology, GLP, Lassa, vaccine, reassortant, virus

## Abstract

**Background:**

Mopeia–Lassa reassortant ML29 virus is an investigational, reassortant virus vaccine for the prevention of Lassa fever caused by Lassa virus (LASV).

**Methods:**

The vaccine virus ML29-SF was prepared in Vero cells using a serum-free culture medium under Good Manufacturing Practice. A 2-week repeat dose toxicity study was performed in guinea pigs under Good Laboratory Practice (GLP) regulations to assess the local and systemic toxicological effects.

**Results:**

Following an intramuscular (IM) or subcutaneous (SC) injection of 10^4^ PFU of ML29-SF LASV vaccine at the start of the study, with a second dose 15 days later, no toxicological response attributable to the vaccine was observed. Vaccine-related effects were not observed in any in-life or post-mortem parameter evaluated, including clinical observations, injection site observations, body temperature, body weight, food consumption, ophthalmology, immunology, hematology, clinical chemistry, gross anatomical pathology, organ weights, and histopathology. An immunogenic response, as measured by the elicitation of IgG antibodies against major LASV immunogens, nucleocapsid and glycoprotein precursor, was observed in all vaccine-treated animals prior to the booster dose (Study Day 15) which endured through the end of the study (Study Day 42). There was no evidence of viral shedding in any vaccinated animal.

**Conclusions:**

Overall, this single-dose vaccine was locally and systemically well tolerated even after a two-dose repeat administration, confirming the high level of safety of ML29-SF vaccination and supporting the future evaluation of this LASV vaccine, including in clinical trials.

## 1. Introduction

LASV causes Lassa fever (LF), a viral hemorrhagic fever, which is responsible for significant morbidity and mortality, with up to 67,000 fatal cases annually [1]. LASV endemic areas cover large regions within West Africa with an at-risk population as high as 200 million people, and evidence indicates that LASV endemic regions are expanding. Except for dengue fever, the estimated global burden of LF is the highest among viral hemorrhagic fevers [2,3]. There is no approved vaccine or specific treatment for LF. LASV genetic diversity is a challenge for vaccine development. Phylogenetically, LASV isolates were initially placed into four major genetic lineages, I–IV [4,5]. Lineages I–III are commonly found in Nigeria, with lineage II south of the Benue and Niger rivers and lineage III to the north [6]. Lineage IV, represented by the reference LASV Josiah strain, occurs across West Africa. Recently, lineages V–VII were also described [4,7,8]. The primary natural reservoir of LASV is the peridomestic multimammate rat, *Mastomys nataliensis* [9]. Other less common rodent hosts include the African wood mouse, *Hylomyscus pamfi,* and the Guinea multimammate mouse, *Mastomys erythroleucus* [10]. Recent detection of LASV in wild monkeys from southern Nigeria and detection of LASV in various domestic animals, chickens, and lizards raises the question of their potential involvement in the transmission and maintenance of LASV in the environment [11,12]. Human infections occur through food contamination or via aerosolization of infected rodent excreta. Human-to-human transmission can occur through contact during care for the sick or deceased [13]. After LASV infection and incubation period of 6–21 days, LF begins with flu-like symptoms of fever, weakness, cough, sore throat, and joint, back, and chest pain [14,15]. In severe cases, this may progress to abdominal pain, vomiting, diarrhea, pharyngitis, and conjunctivitis. Late-stage disease symptoms may include respiratory manifestations and facial edema. Hearing loss can occur during convalescence, even in sub-clinical cases, and is the leading cause of non-congenital sensorineural hearing loss (SNHL) in West Africa [16,17]. Generally, recovery begins 8–10 days after onset; however, a small percentage of patients progress to acute hemorrhagic fever with multi-organ failure [18], with case fatality rates of 1–15% or more [19]. Significant predictors of fatal outcome of LF are hemorrhage, sore throat, and viremia [20]. LASV is an enveloped, single-stranded RNA virus that belongs to the Old-World sub-group of mammalian arenaviruses. The genome comprises two RNA segments, the large (L) and small (S), each containing two genes in an ambisense orientation [21]. The L RNA encodes the largest protein, an RNA-dependent RNA polymerase (L or RdRp), and a matrix-like zinc-binding protein (Z) [22]. The S RNA encodes RNA-associated nucleoprotein (NP) and glycoprotein precursor (GPC). The GPC processes into GP1 (attachment glycoprotein), GP2 (fusion glycoprotein) and SSP (stable signal peptide), forming trimeric spike structures on the virion surface [21]. Coding regions on each RNA segment are separated by an intergenic non-coding region that forms a stable hairpin loop and serves as a transcriptional regulator. Currently, there are no United States Food and Drug Administration (FDA) or European Union Medicines Agency (EMA)-approved LF vaccines or therapeutics, and treatment is limited to supportive care and the off-label use of ribavirin (an antiviral medication). The World Health Organization (WHO) included LASV in the top-priority pathogens list for accelerated vaccine research and development (R&D). In 2017, the WHO released the Target Product Profile (TPP) for an LASV vaccine. According to the WHO TPP, the highest priority is a preventive LASV vaccine for adults and children in LF endemic areas. The vaccine is expected to be an injectable (IM or SC), single-dose formation that induces protection with at least 70% efficacy for 5 years or more from the disease or infection by major LASV genotypes. It is expected to meet WHO pre-qualification criteria, including low cost, thermostability, and environmental and user friendliness.

The experimental ML29 vaccine, one of the promising LF vaccine candidates [23], is a nonpathogenic reassortant virus carrying the L RNA from Mopeia virus (MOPV), and S RNA from LASV. MOPV is an Old-World mammalian arenavirus genetically related to LASV; however, it is nonpathogenic and vaccinates non-human primates (NHPs) against subsequent LASV infection [24]. The ML29 vaccine encodes the LASV/Josiah strain (lineage IV) immunogenic antigens NP and GPC, while the L (RdRp) and Z proteins are derived from nonpathogenic MOPV. Sequence analysis identified ML29-specific mutations that are different from MOPV L and LASV S RNA sequences. In addition, recent studies suggested the mismatch of LASV L and MOPV NP in the viral replication/transcription complex generates truncated RNA species interfering with replication of viral RNA. These features likely contribute to the additional attenuation of ML29 in comparison with parental MOPV [25-27]. In preclinical studies, ML29 vaccine induced protective T-cell immunity against both the homologous LASV/Josiah strain (lineage IV) and the distantly-related Nigerian strain LASV/803123 (lineage II) [28]. The experimental ML29 vaccine was safe, immunogenic, and efficacious in non-human primate (NHP) models, including SIV-infected rhesus macaques mimicking HIV-infected individuals, an important safety advantage of the vaccine considering HIV infection rates among the population in Sub-Saharan Africa [29,30]. ML29 can be manufactured in serum-free medium, and it exhibits multiple features that meet the WHO TPP criteria: (i) an excellent safety profile in all available animal models, including immunocompromised non-human primates mimicking HIV-1 infection; (ii) induction of cross-lineage protective T-cell responses after a single-dose vaccination leading to sterilizing immunity; (iii) genetic stability after serial passages in vitro and in vivo; (iv) efficacy in post-exposure applications; and (v) favorable thermostability [31,32]. Taken together, preclinical data demonstrated that ML29 is immunogenic and efficacious and human clinical trials may be warranted in the future.

To ensure the clinical safety of the vaccine, repeated dose toxicity studies are required to comprehensively evaluate the safety of the vaccines in relevant animal models under controlled conditions and using doses (and dosing regimens) exceeding the clinically intended dose. For single-dose vaccines, repeated dose toxicity studies are standard and pivotal [33,34]. In this study, we performed a preclinical toxicology study in the Hartley guinea pig model to evaluate safety of reassortant ML29-SF experimental LASV vaccine, following Good Laboratory Practice (GLP) and in compliance with 21 CFR Part 58.

## 2. Materials and Methods

### Vaccine and Dosage.

Vaccine ML29-SF was manufactured under Good Manufacturing Practice (GMP), 21 CFR part 110, in Vero cells using a serum-free growth medium formulation. The resulting ML29-SF vaccine virus was purified and concentrated by ultracentrifugation using a 20% sucrose step gradient, resuspended in phosphate-buffered saline PBS, and sterile-filtered using 0.2 μm filter membranes. Sterility and the absence of mycoplasma were confirmed experimentally, and the titer was determined by plaque assay using Vero cell monolayers under agarose overlays. ML29-SF was stored at −80 °C ± 10 °C, protected from light in prefilled 2 mL clear vials each containing 1 mL of vaccine, 1 × 10^5^ plaqueforming units (PFU)/mL, until it was used. Prefilled vials were transferred from the GMP manufacturing facility to Midwest Research Institute MRIGlobal for vaccine administration to guinea pigs. As a control, a “sham” vaccine—an equal volume of 0.9% Physiological Saline, USP (VetOne/Nova-Tech, Inc., Grand Island, NE, USA)—was administered. Before administration, the ML29-SF vaccine was thawed at room temperature or in a roomtemperature water bath. Thawed ML29-SF was stored at 4 °C for no more than 2 h prior to administration. On Study Days 1 and 15, ML29-SF LASV Vaccine or control saline were administered as an IM or SC injection (dose volume 0.1 mL). The 0.1 mL IM dose was administered in a single bolus injection in the left thigh on Study Day 1, and in the right thigh on Study Day 15. The 0.1 mL SC dose was administered in a single bolus injection in the dorsum. Dosages were administered by volume (0.1 mL, constant concentration, target dose 1 × 10^4^ PFU/animal).

### Animal Care and Use.

Animals were used in accordance with the guidelines of the USDA Animal Welfare Act according to IACUC protocol approved by the MRIGlobal Institutional Animal Care and Use Committee (IACUC 23-09, approved 27 July 2023). General procedures for animal care and housing were conducted in accordance with the Guide for the Care and Use of Laboratory Animals. This animal study was conducted according to GLP (21 CFR 58) standards for data collection, quality assurance, and quality control. Since the safety of the ML29 vaccine had been confirmed in earlier studies [28,29] and challenge was not part of this study, early deaths and the removal of animals prior to scheduled euthanasia were not anticipated in this safety study. However, animals were monitored closely for signs including, but not limited, to pain and/or distress; unexplained weight loss (≥20% at any time during the study); moribundity (e.g. lethargy, hunched posture, cool to touch); unresponsiveness; or not eating for a period of time (accompanied by weight loss). Hartley Guinea Pigs (*Cavia porcellus*) were purchased from Charles River Laboratories, Inc. (Saint Constant, Canada). A total of 220 animals (110/sex) were received, of which 210 (105 per sex) were placed on study, with 5 per sex held as alternates (Table 1). Animals were approximately 6–7 weeks of age and weighed between 313.5 and 433.5 g for males and between 323.6 and 404.8 g for females prior to the first dose on Study Day 1 for the Toxicology and Recovery study groups. Animals in the Biodistribution study group were between 373.1 and 440.3 g for males and between 350.6 and 435.8 g for females prior to the first dose on Study Day 1. Upon delivery, animals underwent veterinary examination to ensure they were fit to be placed on study and were acclimated in the MRIGlobal conventional animal facility for approximately 48 h. Body weights were collected during the acclimation period within 3 days of receipt. All animals placed on study were in good health and free of signs of clinical disease at the initiation of dosing. Animals were provided with certified Envigo guinea pig food, ad libitum (Lot Nos. 2040C 062923MA and 2040C 072623MA). Timothy hay or equivalent roughage were supplied daily (Lot Nos. 78098, 77864, and 76263). Diet enrichment with vegetables, such as leafy greens or carrots, was provided in addition to guinea pig feed if there were signs of inappetence. Access to fresh tap water (municipal water supply) or hydrogels was offered ad libitum. The number of animals and dose groups represents a standard design for rodent studies [34], with additional groups appropriately sized to minimize total animal numbers, adhere to blood volume limitation, and achieve study objectives. A statistician was consulted on the study design, who concluded that each group had sufficient statistical justification for the study.

Animals were group-housed upon receipt (through the end of the acclimation period) and moved to individual housing by Study Day −1. Animals were group-housed in larger guinea pig caging and singly housed in smaller polycarbonate cages (Tecniplast, Phoenixville, PA, USA). Animals were single-housed to facilitate clinical observations following dosing, specifically gross motor, and behavioral activity, and to avoid possible influences of group housing (e.g., interaction with cage mates). Animal cages were placed in environmentally controlled rooms, with a temperature of 20 to 25 °C, a relative humidity of 21.2% to 84.7%, and a 12-h light/dark cycle per day, with the exception that the light/dark cycle was interrupted as necessary for protocol-designated procedures (e.g., dosing and observations). Room temperature and humidity were monitored by the OCEAView^®^ monitoring system, and light cycles were monitored by the AmegaView^®^ monitoring system 24 h/day in the conventional animal room. In addition to the food enrichment described above, guinea pig huts and apple wood sticks (BOJAFA Apple Sticks Lot No. X001TUPKBZ, Apple Orchard Sticks Lot. No. 0118JJ and SADA0214) were placed in each cage to provide environmental enrichment for the duration of the study. Animals were randomized (prior to Study Day −1) by weight into the Toxicology, Recovery, or Biodistribution groups, which were sex balanced. Randomization was performed using SAS software (version 9.4) (Cary, NC, USA) using the most recent body weights taken during acclimation. Animals were individually identified using metal ear tags.

### Clinical Observations.

Observations focused on general health were performed at least once daily during the acclimation and pre-dose period and twice daily (at least 4 h apart) starting on Study Day −1 through scheduled euthanasia. Detailed observations were performed at least once prior to Study Day 1, prior to dosing and approximately 1–2 h, 6 h, and 24 h post dose on Study Days 1 and 15, and weekly thereafter (including the day of scheduled euthanasia). These examinations included evaluation of external surface areas, orifices, posture, respiration, and excretory products. During the examination, animals were also observed for common signs including, but not limited to, rough coat, lethargy or increased activity, piloerection, respiratory abnormalities, posture, involuntary motor movements (clonic or tonic), stereotypy, abnormal behavior, gait abnormalities, and vocalizations. Abnormal findings or indications of normality were recorded.

Injection sites were observed for signs of erythema and edema using the Draize dermal irritation scoring system [35] for all Toxicology and Recovery group animals. Animals had dermal observations at the dose site approximately 1–2 h after the dose on dosing days. If erythema or edema was observed, daily dose-site observations continued until the signs resolved.

### Body Temperature.

An Implantable Programmable Temperature Transponder (IPTT; Bio Medic Data Systems, Seaford, DE, USA) was implanted subcutaneously in the dorsal interscapular area under anesthesia. Starting the day following implantation, baseline body temperatures were measured (twice daily) for at least 5 days prior to Day 1 (target Day −5 to Day −1); the average interscapular body temperature for each animal was used as the baseline temperature for that animal. Individual body temperatures were measured and recorded prior to dosing and at 6 h and 24 h post each dose. If any temperature was ≥40.0 °C or ≤36.9 °C, additional measurements were taken and recorded daily until the body temperature returned to normal (37.0 °C to 39.9 °C).

### Body Weights and Food Consumption.

Body weights were recorded during acclimation within 48 h of receipt for randomization, prior to dosing on Study Day 1, weekly for Biodistribution group animals, and twice weekly thereafter for Toxicology and Recovery group animals. A final non-fasted body weight was taken prior to the day of euthanasia. A fasted (overnight) terminal body weight was taken for all animals prior to necropsy. Food consumption was qualitatively assessed daily during morning observations starting on Study Day −2, until termination for all Toxicology and Recovery group animals (except following fasting).

### Ophthalmic Evaluations.

Ophthalmic examinations were performed by a board-certified ophthalmologist once during the pre-dose phase (for Toxicology and Recovery groups, at all dose levels) and again prior to necropsy (Toxicology groups, at all dose levels). Recovery group animals were not examined at the end of the recovery period, given the lack of ML29-SF vaccine-related signs at the end of the dosing phase. The anterior portions of each animal’s eyes and pupillary reflexes were examined using a Welch Allyn Finnoff transilluminator. A mydriatic agent (1% tropicamide ophthalmic solution) was applied to each eye to dilate the pupil. The cornea, aqueous humor, and lens were examined using a slit lamp. The vitreous humor, retina, choroid, and optic disc were examined with an indirect ophthalmoscope and a condensing lens. The examinations were performed in a semi-darkened room.

### Animal Clinical Pathology.

Blood for the clinical pathology evaluations (clinical chemistry and hematology) and immunogenicity evaluation was collected at the times indicated in the Results section. Animals were fasted during the overnight period prior to blood collection the following morning. Whole blood was collected from the cranial vena cava or jugular vein (while under isoflurane anesthesia) into an EDTA anticoagulant tube or a serum separator tube. Immediately following collection, all anticoagulant tubes were gently inverted several times to sufficiently mix the blood and anticoagulant. EDTA tubes were stored at 4 ° C. For hematology, multiple parameters (Table 2) were measured on a Siemens Advia 2120 Hematology analyzer. Serum was allowed to clot for at least 30 min and centrifuged at 2400× *g* for 10 min at 4 °C. For clinical chemistry, multiple parameters (Table 2) were measured on a Beckman Coulter AU680 at Antech Diagnostics GLP. Continuous group mean data examined statistically, separately for males and females (e.g., body weight, body temperature, mean clinical pathology parameters, and organ weights) were evaluated (SAS software; version 9.4) using a one-way analysis of variance (ANOVA) [36], a repeated measures ANOVA, and/or using the Dunnett’s Test [37] if the ANOVA indicated significance. In general, statistical tests were performed as two-tailed tests taken as significant with (*p*) levels of <0.05.

### Enzyme-Linked Immunosorbent Assay (ELISA).

Immunogenicity (seroconversion) was used to confirm the vaccination of animals subject to safety analysis. Serum samples were stored at −80 °C prior to being thawed for testing. The anti-LASV IgG ELISA kits, Zalgen ReLASV Lineage IV Linked GP IgG ELISA kit (Cat. # 10732) and ReLASV Lineage IV Nucleoprotein (NP) IgG ELISA Kit (Cat. # 10730) were used to quantify immunoglobulin subtype G (IgG) antibodies against lineage specific LASV antigens using an ELISA, in which purified recombinant LASV reference protein (GPC or NP) was used as the solid-phase immobilized antigen and an enzyme-conjugated anti-gamma chain secondary antibody was used as the reporter. The assay was performed in accordance with the manufacturer’s instructions, except that the human-conjugated secondary antibody was replaced with a guinea pig-conjugated antibody.

Briefly, diluted samples and control sera starting from 1:100 dilution were incubated in the wells of 96-well plates that had been pre-coated with recombinant LASV GP or NP antigen. The anti-LASV antibodies present in the samples were allowed to bind to the LASV antigen. After washing, the bound anti-LASV antibodies were detected by an anti-guinea pig IgG horseradish peroxidase (HRP) conjugate, followed by the addition of a peroxidase substrate. Plates were read on a microplate reader (BioTek, Winooski, VT, USA) at 450 nm with a reference of 650 nm. The optical density (OD) values were exported, and analysis was performed using Microsoft Excel, version 2408. The reportable value for each sample is the concentration of anti-LASV IgG antibodies in μg/mL reported from a 4-parameter curve fit of the positive control mean OD and IgG concentration. Statistical analysis was performed with GraphPad Prism version 10 (GraphPad Software, Boston, MA, USA).

### Quantitative Polymerase Chain Reaction (qPCR).

On Study Days 2, 7, 17, 28, and 42, oral swabs, urine, and fecal samples were collected to evaluate the shedding of the vaccine virus. Guinea pig swab and urine samples (0.2 mL) were extracted using the IndiSpin QIAcube HT Pathogen Kit. Fecal samples were homogenized using the Miltenyi GentleMACS in phosphate-buffered saline (PBS) and then aliquoted to prepare a 0.2 mL sample. Guinea pig fecal, swab (0.2 mL), and urine samples (0.2 mL) were extracted using the IndiSpin QIAcube HT Pathogen Kit. PBS was used as a negative control. A positive control was prepared by diluting ML29-SF vaccine stock in PBS. The ML29-SF RNA was quantified using quantitative reverse transcriptase- (qRT)-PCR on a QuantStudio 6 Flex Real-Time PCR System with the LASV-specific primers and probe (Table 3). An 8-point standard curve using a qualified reference standard (RS) dilution series comprising synthetic RNA with sequence specific to the LASV gene target amplicon (Table 3) was used to determine the PCR concentration (genome copies (gc)/mL or gc/g) of ML29-SF RNA in each test sample.

## 3. Results

### 3.1. Study Design

Previous studies in guinea pigs and NHPs using a single 10^3^ PFU dose, SC route, indicated that the experimental vaccine was safe and elicited complete protection in vaccinated animals against lethal LASV challenge [28-31]. The objective of this study was to assess the safety and determine the potential toxicity, local tolerance, immunogenicity, and biodistribution profile of the experimental ML29-SF LASV vaccine when administered to Hartley guinea pigs via the IM or SC route on Study Day 1 and 15 (2 total doses, 10^4^ PFU/dose), as well as to determine whether persistence, delayed toxicity, and/or recovery occur after a minimum 28-day recovery period. The repeat (two-dose) toxicity study design is standard for single-dose vaccines to confirm their safety [34]. The dose 10^4^ PFU was chosen based on the previous efficacy studies, including in guinea pigs [28,29].

The guinea pig is a species accepted by regulatory authorities for these types of experiments and the study protocol was based on work utilizing guinea pigs in previous studies. Additionally, a large database exists for this species and Hartley strain, which allowed for comparisons with previous data. A total of 210 (105/sex) Hartley guinea pigs were used in this study. The first day of dosing was designated Study Day 1. The initiation of dosing was separated into Toxicology and Recovery study groups, comprising equal numbers of males and females (n = 60 each), and a Biodistribution study group also comprising equal numbers of males and females (n = 45 each) (Table 1). A clinical evaluation of the animals was performed to evaluate the health of the animals prior to the first day of dose administration. All animals were in good health and free from any signs of clinical disease or behavioral abnormalities that would interfere with the study.

In the Toxicology group, male and female guinea pigs (10 animals/sex/group; 4 groups) were administered ML29-SF LASV Vaccine or control article as an IM or SC injection on Study Days 1 and 15 (Table 1). Animals were euthanized on Study Day 17 for examination. In the Recovery group, male and female guinea pigs (5 animals/sex/group; 4 groups) were administered control article or ML29-SF LASV Vaccine as an IM or SC injection on Study Days 1 and 15 (Table 1), followed by a recovery period of 4 weeks. Animals were euthanized on Study Day 42. The examined parameters included mortality and moribundity checks, clinical observations (including Draize scoring of the dose site), body temperature, body weight, food consumption, ophthalmology, clinical chemistry, hematology (Table 2), immunology, gross pathology, organ weights, and microscopic pathology. In the Biodistribution group, male and female guinea pigs (15/sex/group; 3 groups; no SC control group) were administered control article or ML29-SF LASV Vaccine as an IM or SC injection on Study Days 1 and 15 (see Table 1). Guinea pigs were sampled for viral shedding and biodistribution evaluation using qPCR (Table 3). Animals were euthanized on Study Days 2, 7, 17, 28, and 42 (3/sex/group/day). The examined parameters included mortality and moribundity checks, clinical observations, body weight, and viral shedding.

### 3.2. Clinical Observations

No changes in the clinical observational profile attributable to the ML29-SF vaccination was observed. No changes at the dose site were observed either (Table 4). Control groups included PBS-inoculated animals using the same administration routes as test groups. Positive control was not included as this is not required by guidance [34]. Clinical observations appeared to be similar to controls and indicative of the typical background for otherwise normal guinea pigs. Most of the clinical signs were associated with the skin, including (but not limited to) scabbing/redness around the injection sites, hair loss, trace of blood at the injection site, and minimal rough coat.

There were no ophthalmic findings attributable to the ML29-SF vaccination based on the incidence of lesions observed, the distribution between groups, and the comparison between the pre- and post-exposure findings (Table 4).

### 3.3. Body Temperature, Weight, and Food Consumption

**Figure 1.**
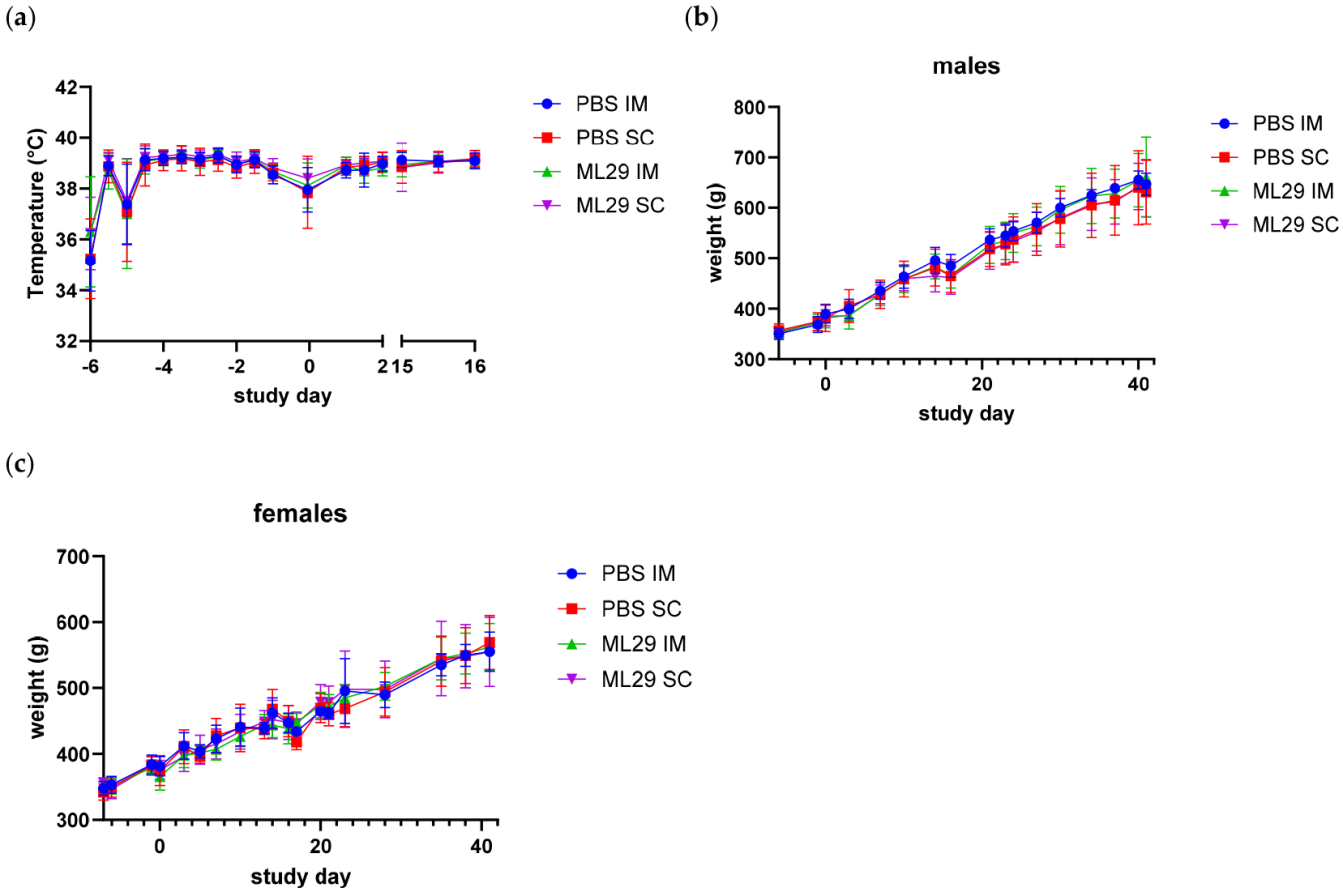
Guinea pigs’ body weight and temperature after ML29-SF administration. (**a**) Body temperature (combined males and females). Study groups are indicated on the right. (**b**) Body weights in PBS control- and vaccine-treated groups, males, and (**c**) body weights in females. Study groups are indicated on the right.

Body temperature change over the study interval was generally similar between the controls and the ML29-SF vaccinated animals. Lower temperatures at the beginning of the study are likely attributable to the combination of the stress from transportation, mild procedural hypothermia from the anesthetic event for transponder insertion, or the transponder not having time to reach body temperature. Similarly, a mild decrease in the body temperature was detected during vaccine administration, although it was comparable across all groups, including negative controls. The temperatures were similar in the males and females; therefore, the data for both sexes and both the Toxicology and Recovery study groups were combined (Figure 1a). In summary, no body temperature changes attributable to the vaccine administration were observed.

Body weight change over the study interval was generally similar between controls and ML29-SF treated animals. As expected, males had a larger body mass than females; therefore, body weights are presented separately for males and females to improve clarity (Figure 1b,c). Food consumption remained unchanged after the vaccinations, and no food consumption changes attributable to the ML29-SF vaccination were observed (Table 4). Any differences (e.g., slower eating compared to control animals) were well within a typical range for guinea pigs of the same breed, age, and weight used.

### 3.4. Clinical Chemistry, Hematology, and Pathology

Guinea pigs are an FDA-acceptable and widely used small animal model of LF disease, sharing some hallmarks of fatal LASV infection in humans and NHPs, including high viremia, leukopenia, thrombocytopenia, and markers of liver dysfunction. In clinical trials, elevated levels of AST in plasma and high viremia were strongly associated with death [38]. These findings were reproducible in NHP [39-41] and in guinea pigs [42-44], including animals infected with a lethal dose of LASV or with LCMV-WE, a surrogate model of LASV infection [45]. Renal pathologic features, high creatinine, and urea blood levels were notable clinical features in LF patients in Nigeria [46]. In guinea pig models, renal function abnormalities, albeit to a lesser extent, manifested as a transient increase in nitrogen levels in urine [44]. In this study, hematology, blood, and urine chemistry were evaluated with special attention paid to markers of fatal LF in experimental animals and in LF patients to access the potential toxicity of ML29-SF in the Hartley guinea pigs model.

**Figure 2.**
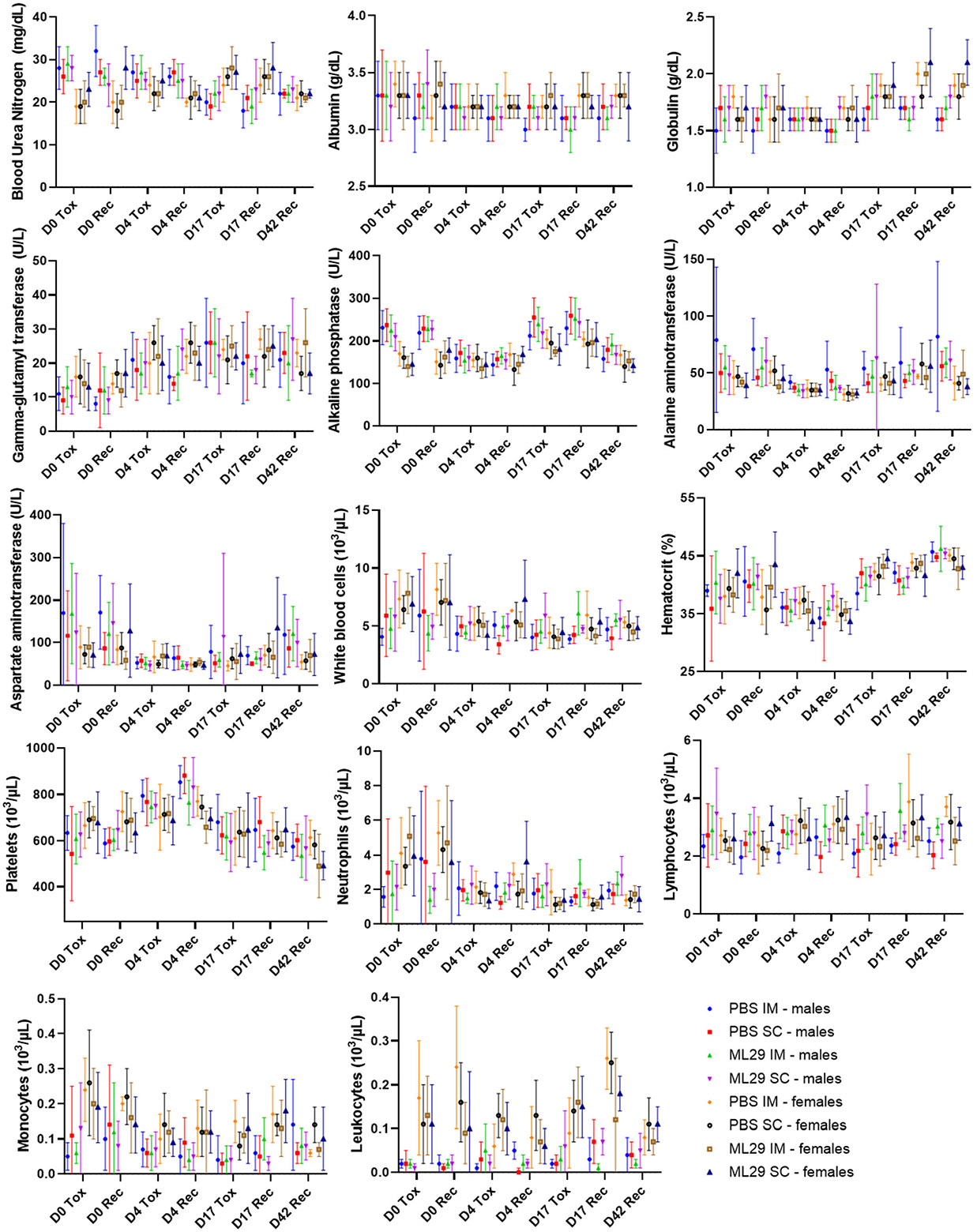
Clinical chemistry and hematology parameters in vaccinated guinea pigs. Chemistry or hematology parameters are shown on the Y axis. For each parameter, groups are shown on the X-axis. Error bars show standard deviation.

As seen in Figure 2, after administration of ML29-SF, via either the SC or IM routes (Groups 3 and 4), all hematology and clinical chemistry markers fluctuated within normal ranges and no correlation with vaccine administration or vaccine route was observed. All other blood chemistry and hematology markers for animals from the experimental and control groups were in ranges described for healthy guinea pigs, accounting for sex and age (Table 4) [47]. In summary, no changes in any clinical chemistry or hematology parameters observed in this study were attributable to ML29-SF administration.

In patients and experimentally infected animals, LASV-specific gross anatomical pathology and histopathology findings are rarely associated with fatal outcomes. In small animal models, strain 13 and Hartley guinea pigs, pathology findings associated with LF disease progression included interstitial inflammation, edema in lungs, and fatty steatosis in liver. In this study, there were no abnormal gross pathology findings after ML29-SF administration (change in organ weight or organ specific tissues findings attributable to ML29-SF vaccination). No histologic evidence of differences between ML29-SF-treated and control groups was observed, either at the end of the dosing phase (Toxicology Group-Day 17) or at the end of the recovery phase (Recovery Group-Day 42). Additional in-depth histopathological analysis will be carried out in the future. In endemic areas of West Africa, LASV infection can cause conjunctivitis in acutely infected patients and this LASV-induced ophthalmic pathology can be modeled in guinea pigs [48]. We assessed the ophthalmic status of guinea pigs after ML29-SF administration as an additional marker of safety/toxicity for live LF vaccines. No ophthalmic changes associated with ML29-SF administration were observed in this study.

In general, apparent changes were indicative of normal variation, based on a relatively small magnitude of change, lack of dose response, and lack of correlated underlying gross or microscopic pathology findings.

### 3.5. Biodistribution and Viral Shedding

Viremia in progressed LF patients and high virus titters in the urine of naturally infected rodents are crucial features of LASV infection in humans and natural hosts, respectively. Therefore, ML29-SF viremia was assessed by the detection of viral RNA (RNA-emia) using a highly sensitive qRT-PCR assay. As controls for qRT-PCR assay, synthetic RNA (Table 3) and RNA isolated from ML29-SF vaccine virus were used. All the tested serum samples were negative for the LASV S segment target. Tissue samples were collected for potential biodistribution analysis (RT-qPCR). No evidence of viral RNA presence in blood or viral shedding in oral swabs, urine, or feces (Table 4) was indicated in any control or treated (RT-qPCR) animal following IM or SC administration of the ML29-SF LASV vaccine at any timepoint tested over the in-life and recovery phases (Study Days 2, 7, 17, 28, and 42).

### 3.6. Immunogenicity

**Figure 3.**
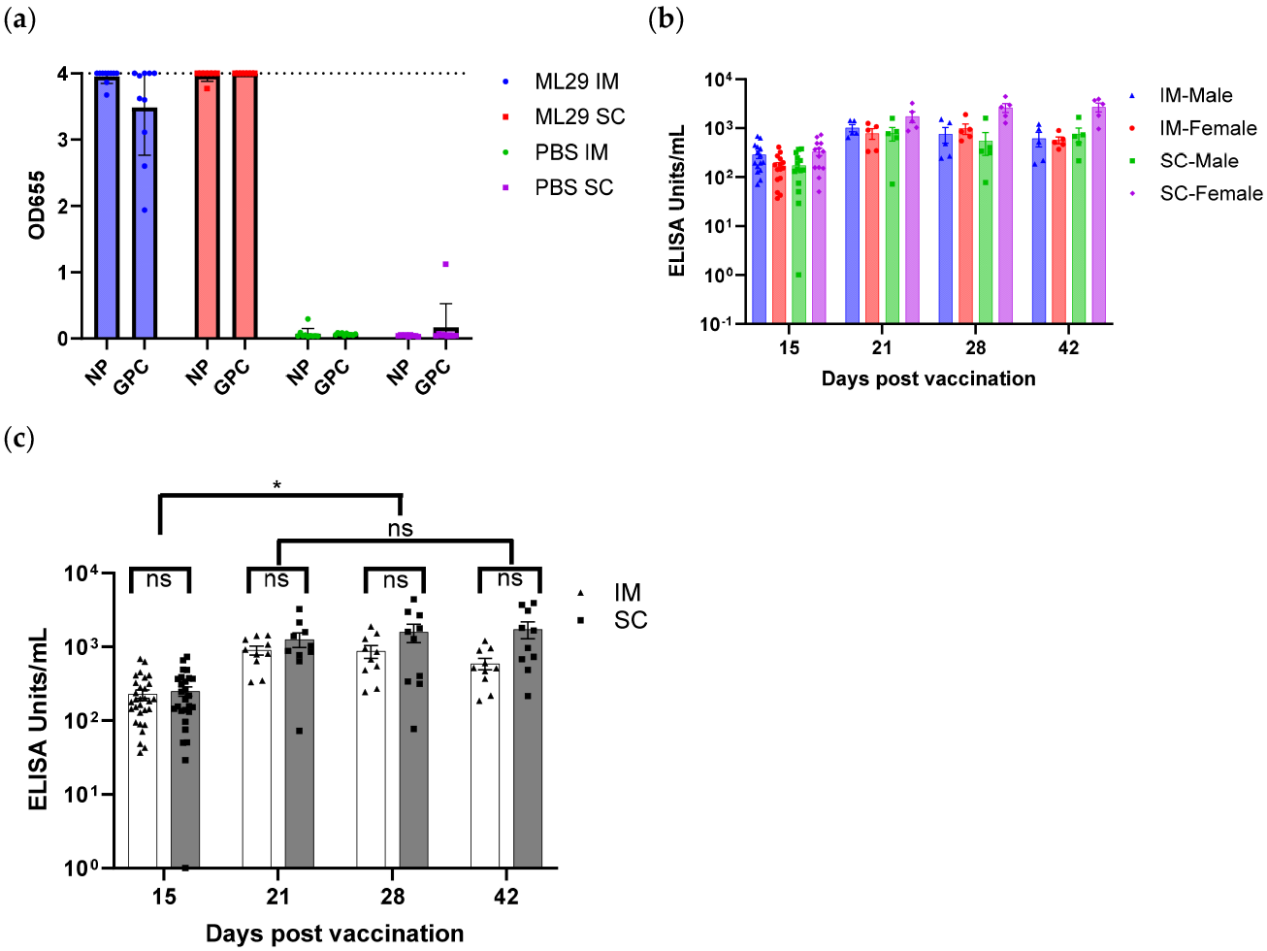
LASV-specific IgG response after ML29-SF administration. (**a**) Antibody responses to GPC and NP antigens on day 42 after vaccination. Guinea pigs received 10^4^ PFU on days 1 and 15 IM or SC as indicated on the right. Controls similarly received PBS as placebo vaccination. On the X-axis, antigen kit is indicated (NP- or GPC-specific). (**b**) Serum antibody response to NP in vaccinated guinea pigs, by immunization route (IM and SC) and sex (males/females); (**c**) Mean serum antibody response in guinea pigs vaccinated via IM and SC routes. One-way repeated measures ANOVA; * *p* < 0.0001; ns, not significant. ELISA Units/mL indicate relative units in comparison with reference NP antigen.

Serum antibody responses were determined to confirm the exposure of animals to the vaccine and the magnitude of humoral response to the vaccine. An immunogenic response was observed in all Toxicology and Recovery group animals following IM or SC administration of the ML29-SF LASV vaccine, including on Study Day 15 in Toxicology group animals and Study Day 15 through Study Day 42 in Recovery group animals. Initially, we determined antibody responses to both GPC and NP antigens on day 42 (Figure 3a). For this first test, we screened 37 serum samples in duplicate. Antibodies were detected to both antigens in the vaccine groups, confirming that both GPC and NP within the ML29-SF vaccine are immunogenic in guinea pigs. At 1:100 dilution, the antibodies showed maximal OD655 signal, indicating the high immunogenicity of both antigens. As expected, no immunogenic response attributable to the ML29-SF LASV vaccine was noted in the control animals.

Next, ELISA was performed using optimized conditions. LASV GPC antigen is a difficult antigen to consistently measure LASV-specific antibody responses for several reasons, including that it is immunogenic predominantly in its metastable pre-fusion configuration, and that anti-GPC antibodies appear at late stages of infection or immunization [49]. LASV NP is the predominant antigen in virions and infected cells, is detected at an early stage of the infection, and is metabolically stable. Therefore, for the remaining sera, we evaluated IgG responses to LASV NP antigen only (Figure 3b,c) and expressed in relative ELISA Units/mL, according to the NP reference and NP ELISA kit instructions. Overall, antibody responses were significantly elevated (mixed-effects model, *p* < 0.0001) at 21, 28, and 42 days compared to 15 days after vaccination. The application of a mixed-effect model to examine the interaction of time, sex, and route of administration (IM vs. SC) did not reveal a significant difference in any single factor.

## 4. Discussion

Changing ecology, climate, international commerce, travel, and other factors have the potential to contribute to the spread of viruses and their natural vectors, increasing the chances of disease outbreaks and epidemics including LASV [50,51]. When the rapid containment of disease outbreaks is needed, live virus vaccines have the advantage of eliciting rapid, robust immunity with a single vaccination. Epidemiological studies in West Africa indicate that a live vaccine is the most feasible approach to control LF [2], and there is renewed interest in live vaccine approaches [52]. The only arenaviral vaccine approved for human use, live-attenuated vaccine Candid 1, effectively reduced cases of Argentine hemorrhagic fever in endemic areas [53,54]. Live virus vaccines are among the most costeffective and broadly used public health interventions, representing approximately half of all licensed vaccines. In recent years, live vaccines Zostavax, FluMist, Rotarix, Ixchiq, and others have been approved for human use, demonstrating that live vaccines meet stringent FDA safety standards. Pediatric FluMist and Rotarix vaccines were designed using reassortant technology. Reassortment was also used to generate the experimental ML29 vaccine seed. This technology is applicable for viruses with segmented RNA genomes. The aim of this technology is to prepare a reassortant live virus carrying genes or RNA segments encoding RdRp from a nonpathogenic virus-donor along with genes needed for immune response and protection from the pathogenic virus.

In line with the results of previous LCMV reassortant experiments [55,56], a recent reverse genetics study provided direct evidence that the L gene encoding RdRp was responsible for pathogenic features of LASV [57]. In the ML29 reassortant, the nonpathogenic phenotype and safety are mainly associated with MOPV L RNA encoding RdRp [26]. Notably, in areas of MOPV circulation, there are no clinical cases of LF, and experimentally MOPV-vaccinated monkeys were fully protected against fatal LASV challenge [58]. Advantages of reassortant ML29-SF vaccine include expression of both LASV GPC and NP as major immunogens, cross-protection from distinct phylogenetic lineages, as well as resistance to reversion of reassortant technology, high yields during manufacturing, and low effective dose [31].

Strong anti-NP immunity at an early point in infection is essential for the effective control of viral replication. The recombinant LCMV vector lacking NP failed to elicit detectable CD8+ T-cell responses unless NP was trans-complemented [59]. Likewise, a measles vector (MV), simultaneously expressing LASV GPC and NP, was more effective than an MV expressing LASV GPC alone [60]. In earlier studies, the ML29 vaccination induced LASV-specific cross-protective responses [28] with immune profiles mimicking the long-term immunity induced by naturally attenuated LASV- or MOPV-like viruses [61-63]. Transcriptome profiling of human peripheral mononuclear cells (hPBMC) from healthy donors exposed to ML29 and MOPV revealed that gene expression patterns in control (media exposed) and ML29-exposed hPBMC clustered together and differed from MOPV-exposed hPBMC, further confirming the safety profile of ML29 [29]. Notably, transcriptome profiling of hPBMC showed the most striking differences in the expression profiles of interferon-stimulated genes (ISGs), as well as genes involved in apoptosis, in NF-kB signaling, and in the coagulation pathways as early as the first 24 h after exposure with LASV and ML29 [30]. This suggests that balanced activation of innate immunity resulted in effective adaptive immune responses that occurred at a very early stage after the ML 29 vaccination. The ability of ML29 to quickly develop effective protective immunity is crucial for emergency settings when the vaccine is intended for the protection of at-risk persons in an ongoing outbreak [64].

In LASV endemic areas, the SNHL remains the most prominent cause of morbidity in survivors, affecting up to 30% of convalescent patients. There is a potential concern that the immune response caused by immunization with LASV vaccines could trigger SNHL. This concern was recently addressed during the international webinar “SNHL, Lassa virus disease and vaccines” [65]. A strong reduction in the viral load and rapid viral clearance after challenge of vaccinated animals indicates that vaccination would reduce the risk of virus-related immunopathology. No vaccine-associated SNHL or autoimmune signals have been recorded in clinical trials to date. Likewise, hearing tests conducted by acoustic startle in immunocompromised STAT-1−/− mice (a small animal model to study LASV-induced SNHL) immunized with ML29 adjuvated with defective interfering particles did not detect any measurable hearing loss during the observation period (62 days) [27].

Several other live vaccines for the prevention of LF are in development, including vesicular stomatitis virus (VSV)-, adenovirus (AdV)-, measles (MeV)-, rabies-, and MOPV-vectored vaccines [66-73]. Candidate DNA and mRNA vaccines have been described [74,75]. In the case of viral vaccine vectors, it is often the viral vector, not the vaccine-relevant antigens expressed by these vectors, that are the predominant immunogens and drivers of the immune responses. Thus, anti-vector immunity and, in some cases, vector safety concerns, are among the limitations of viral-vectored vaccines [76].

The objective of this study was to determine the potential toxicity, local tolerance, humoral immunogenicity, and biodistribution profile of the ML29-SF LASV vaccine in a guinea pig model under GLP, as required by FDA before testing experimental vaccines in clinical trials. The results showed that after IM or SC treatment with ML29-SF, no toxicological responses attributable to ML29-SF were observed. The potential pathological markers of arenaviral infection in guinea models were also examined. Vaccination-related adverse reactions were not observed in any in-life or post-mortem parameter evaluated, including clinical observations, dose-site observations, body temperature, body weight, food consumption, ophthalmology, immunology, hematology, clinical chemistry, gross pathology, and histology. These results suggests that both IM and SC routes are safe, even at a repeated 10^4^ PFU per dose ten times greater than an expected human dose. An immunogenic response was observed in all treated Toxicology and Recovery group animals (Study Day 15 through 42), indicating successful dosing. Based on the RT-qPCR results, viral shedding was not indicated in any vaccinated animal. Additional studies are needed to confirm these findings, especially for potentially low-titer, transient shedding. The vaccine was locally and systemically well tolerated. GLP efficacy was not performed in this study and will be designed based on earlier preclinical experiments [28,29] including studies of T-cell response and cytokines. Taken together, nonclinical research including this study indicated that the reassortant LF vaccine is safe, genetically stable, highly immunogenic, and protective with a single dose, supporting further development of the vaccine.

## Figures and Tables

**Table 1.** Group Designation and Dose Levels.

Group Number	Test Article	Target Dose (PFU) ^1^	Route	Injection Day(s)	Animal Designation and Number					
					Toxicology ^2^		Recovery ^3^		Biodistribution	
					Male	Female	Male	Female	Male	Female
1	Control (PBS)	0	IM	1 and 15	10	10	5	5	15	15
2	Control (PBS)	0	SC	1 and 15	10	10	5	5	--	--
3	ML29-SF LASV Vaccine	1 × 10^4^	IM	1 and 15	10	10	5	5	15	15
4	ML29-SF LASV Vaccine	1 × 10^4^	SC	1 and 15	10	10	5	5	15	15

1 The dose volume was 0.1 mL (based on the concentration of the ML29-SF) and administered in a single injection for both the intramuscular (IM) administration and the subcutaneous (SC) administration on Days 1 and 15.

2 Necropsy Day 17.

3 Necropsy Day 42.

**Table 2.** Hematology (left column) and Clinical Chemistry (right column) Parameters.

Hematology	Clinical Chemistry
cell morphology ^1^	albumin (Alb)
total leukocyte count (WBC)	A/G ratio (A/G)
neutrophils (NEU)	aspartate aminotransferase (AST)
lymphocytes (LYM)	total protein (T-Prot; TPRO)
monocytes (MONO)	gamma glutamyl transpeptidase (GGT)
basophils (BAS)	glucose (Gluc, GLU)
eosinophils (EOS)	triglyceride (Trig)
% neutrophils (NEU%)	creatinine (Creat, CREA)
% lymphocytes (LYM%)	potassium (K)
% monocytes (MON%)	calcium (Ca)
% basophils (BAS%)	creatine kinase (CK, CPK)
% eosinophils (EOS%)	globulin (Glob)
erythrocyte count (RBC-Red Blood Cell)	alanine aminotransferase (ALT)
hemoglobin concentration (HGB, HB)	alkaline phosphatase (ALP)
hematocrit (HCT)	total bilirubin (T-Bili, TBIL)
mean corpuscular volume (MCV)	direct bilirubin (D-Bili, DBIL)
mean corpuscular hemoglobin (MCH)	cholesterol (Chol)
mean corpuscular hemoglobin concentration (MCHC)	urea nitrogen (UN)
platelet count (PLT)	sodium (Na)
mean platelet volume (MPV)	chloride (Cl)
red blood cell distribution width (RDW%)	phosphorus (Phos, P)
reticulocytes (% and ABS, RET and RET%)	

1 Manual reading of smears for morphology check was only carried out as necessary.

**Table 3.** LASV S Segment Primer, Probe, and Synthetic RNA Sequences.

Forward Primer (5’ to 3’)	TCCAACATATTGCCACCATC
Probe (5’ to 3’)	FAM-TGCCTTCACAGCTGCACCCA-MGB
Reverse Primer (5’ to 3’)	GCTGACTCAAAGTCATCCCA
Synthetic RNA (5’ to 3)’	GGGUUGAUUGUCUCCAACAUAUUGCCACCAUCCAGCAUGCAAGCUCCUGCCUUCACAGCUGCACCCAAGCUAAAAUUAUAACCUGAGAUAUUCAAAGAGCUUUUCUUGGUGUCAAUCAUAUUUAGGAUGGGAUGACUUUGAGUCAGCCUGUCUAAG

**Table 4.** Summary of observations.

Group Number	Treatment, Route	Mortality/Morbidity	General Health	Ophthalmology	Food Consumption	Organ Weights	Hematology	Clinical Chemistry	Shedding *
1	Control PBS, IM	None	Healthy	No Change	Normal	Normal	No Change	No Change	ND
2	Control PBS, SC	None	Healthy	No Change	Normal	Normal	No Change	No Change	ND
3	ML29-SF LASV Vaccine, IM	None	Healthy	No Change	Normal	Normal	No Change	No Change	ND
4	ML29-SF LASV Vaccine, SC	None	Healthy	No Change	Normal	Normal	No Change	No Change	ND

* ND, not detected; control RNA isolated from ML29-SF vaccine was detectable.

## Data Availability

The data presented in this study are available on request from the corresponding authors due to non-disclosure agreement.

## Abbreviations

The following abbreviations are used in this manuscript:

CFR: Code of Federal Regulations
CRO: Contract Research Organization
CDMO: Contract Development and Manufacturing Organization
ELISA: Enzyme-Linked Immunosorbent Assay
EMA: European Medicines Agency
FDA: Food and Drug Administration
HRP: Horseradish Peroxidase
IM: Intramuscular
GLP: Good Laboratory Practices
GMP: Good Manufacturing Practices
GP: Glycoprotein
GPC: Glycoprotein Precursor
LASV: Lassa Virus
LCMV: Lymphocytic Choriomeningitis Virus
LF: Lassa Fever
MOPV: Mopeia Virus
NHP: Non-Human Primates
NP: Nucleoprotein
PCR: Polymerase Chain Reaction
qPCR: Quantitative Polymerase Chain Reaction
qRT-PCR: Quantitative Reverse Transcriptase-PCR
RdRp: RNA-dependent RNA polymerase
SC: Subcutaneous
SSP: Stable Signal Peptide
TPP: Target Product Profile
USDA: United States Department of Agriculture
WHO: World Health Organization
